# Validation of the BOADICEA model for epithelial tubo-ovarian cancer risk prediction in UK Biobank

**DOI:** 10.1038/s41416-024-02851-z

**Published:** 2024-09-18

**Authors:** Xin Yang, Yujia Wu, Lorenzo Ficorella, Naomi Wilcox, Joe Dennis, Jonathan Tyrer, Tim Carver, Nora Pashayan, Marc Tischkowitz, Paul D. P. Pharoah, Douglas F. Easton, Antonis C. Antoniou

**Affiliations:** 1 Centre for Cancer Genetic Epidemiology, Department of Public Health and Primary Care, University of Cambridge, Cambridge, UK; 2 Department of Medical Genetics, NIHR Cambridge Biomedical Research Centre, University of Cambridge, Cambridge, UK; 3 Department of Computational Biomedicine, Cedars-Sinai Medical Center, Los Angeles, CA USA; 4 Centre for Cancer Genetic Epidemiology, Department of Oncology, University of Cambridge, Cambridge, UK

**Keywords:** Genetic counselling, Ovarian cancer, Risk factors, Epidemiology

## Abstract

**Background:**

The clinical validity of the multifactorial BOADICEA model for epithelial tubo-ovarian cancer (EOC) risk prediction has not been assessed in a large sample size or over a longer term.

**Methods:**

We evaluated the model discrimination and calibration in the UK Biobank cohort comprising 199,429 women (733 incident EOCs) of European ancestry without previous cancer history. We predicted 10-year EOC risk incorporating data on questionnaire-based risk factors (QRFs), family history, a 36-SNP polygenic risk score and pathogenic variants (PV) in six EOC susceptibility genes (*BRCA1*, *BRCA2*, *RAD51C*, *RAD51D*, *BRIP1* and *PALB2*).

**Results:**

Discriminative ability was maximised under the multifactorial model that included all risk factors (AUC = 0.68, 95% CI: 0.66–0.70). This model was well calibrated in deciles of predicted risk with calibration slope=0.99 (95% CI: 0.98–1.01). Discriminative ability was similar in women younger or older than 60 years. The AUC was higher when analyses were restricted to PV carriers (0.76, 95% CI: 0.69–0.82). Using relative risk (RR) thresholds, the full model classified 97.7%, 1.7%, 0.4% and 0.2% women in the RR < 2.0, 2.0 ≤ RR < 2.9, 2.9 ≤ RR < 6.0 and RR ≥ 6.0 categories, respectively, identifying 9.1 of incident EOC among those with RR ≥ 2.0.

**Discussion:**

BOADICEA, implemented in CanRisk (www.canrisk.org), provides valid 10-year EOC risks and can facilitate clinical decision-making in EOC risk management.

## Introduction

Epithelial tubo-ovarian cancer (EOC) is the seventh most common cancer and the eighth leading cause of death from cancer in women globally [1]. The 5-year survival rate drops dramatically as the stage of diagnosis advances, from 95% in Stage 1 to 15% in Stage 4 [2]. Over 75% of EOC are diagnosed at an advanced stage, due to a lack of symptoms at the early stage of the disease [3, 4]. Although population-based screening with CA-125 testing and transvaginal ultrasonography has limited efficacy [5], prevention options such as risk-reducing bilateral salpingo-oophorectomy [6, 7] can reduce the EOC risks but associated with adverse effects [8–10]. Cancer risk prediction models have the potential to identify women at high risk who are most likely to benefit from such interventions.

The multifactorial Breast and Ovarian Analysis of Disease Incidence Algorithm (BOADICEA), implemented in the CanRisk web tool (www.canrisk.org), incorporates the simultaneous effects of genetic risk factors including rare pathogenic variants (PVs), common genetic variants summarised as a polygenic risk score (PRS), pedigree-structured family history of breast, ovarian, prostate and pancreatic cancer, a residual polygenic component to account for the unexplained familial aggregation and established questionnaire-based risk factors (QRFs). It also includes demographic factors including age, year of birth and country of residence in risk prediction [11]. There are two variants of BOADICEA which have been optimised to model separately the risk of breast and ovarian cancer. For EOC, BOADICEA incorporates PVs in six susceptibility genes *BRCA1, BRCA2, RAD51C, RAD51D, BRIP1* and *PALB2* and a 36-SNP PRS [12]. The QRFs include height, body mass index (BMI), parity, use of oral contraceptive and menopausal hormone therapy (MHT), endometriosis, and tubal ligation.

One study previously assessed an earlier version of BOADICEA for predicting EOC [13], using data from a relatively small sample of 1961 women (374 incident EOCs) from the UK Collaborative Trial of Ovarian Cancer Screening (UKCTOCS). The study assessed only the 5-year EOC predicted risks, no information was available on the PVs in the six EOC susceptibility genes and the PRS used was limited to a subset of 15 SNPs [13]. Here, we evaluate for the first time the full multifactorial BOADICEA model in predicting the 10-year EOC risk prediction in the UK Biobank cohort of 199,429 women.

## Methods

### Study population: UK Biobank

UK Biobank recruited more than 500,000 participants (273,325 females) from the UK aged 40–69 at recruitment, which was from 2010 to 2016 [14]. A wide range of lifestyle and hormone-related phenotypic variables were collected at baseline. Summary family history of breast and prostate cancer among first-degree relatives was available and was used to construct the pedigree format family history used in BOADICEA (details in Supplementary Material). UK Biobank did not collect information on ovarian cancer family history. Information on cancer diagnoses (coded as ICD10) and deaths was obtained through linkages from national cancer and death registries. The most recent update of patient information in the UK Biobank prior to this study was on June 1, 2022, which was used as the last follow-up date. The 36-SNP PRS [12] was constructed using genotyped and imputed data (details in Supplementary Material). We considered PV in *BRCA1, BRCA2, RAD51C, RAD51D, BRIP1* and *PALB2* if there were any frameshift, nonsense, canonical splice site variants or large genomic deletions except those in the last exons or the last 50 bp of the penultimate exons from the whole exome sequencing data. We restricted our analysis to unrelated women of European ancestry, based on genetic kinship, with no personal history of cancer or bilateral oophorectomy at entry (Supplementary Fig. 1). All participants in the UK Biobank provided informed consent. UK Biobank has approval from the North West Multi-centre Research Ethics Committee (MREC) as a Research Tissue Bank (RTB) approval.

### EOC risk prediction

We predicted the EOC risk up to 10 years using BOADICEA [11] with age- and calendar period-specific UK population incidences. To avoid including potential prevalent cases, we considered follow-up to start at the date of recruitment plus one year. The follow-up was censored at the date of cancer diagnosis, oophorectomy, death, 10-year follow-up, date of last linkage or age 80, whichever occurred first. For unaffected women with shorter follow-up time (<10 years), we predicted the EOC risk to the censoring age.

### Statistical analysis

We quantified the overall model calibration using the ratio of the expected (E) to observed (O) number of incident EOC cases overall and by calibration plots, considering the deciles of predicted risks. We also calculated the calibration slope by fitting a logistic regression with the observed cancer outcome (affected/unaffected) as the dependent variable and the log-odds of individual predicted risk as the independent variable [15]. We assessed the model discriminative ability in terms of the area under the receiver operating characteristic curve (AUC) and the Harrell’s concordance index (C-index) [16]. We assessed the model performance using combinations of included risk factors, separately in women aged younger and older than 60 years and separately for carriers of PVs in any of the six genes considered in the model.

We investigated risk classification by calculating the proportion of all study participants and EOC cases in different risk groups, using both age-independent and age-dependent 10-year absolute risk thresholds based on relative risk (RR) thresholds as described by Pashayan et al [17]. Previous studies suggested that women with lifetime EOC risk from age 20 to 80 less than 3.5% to be considered as at “low risk”, 3.5–5% as at “average risk”, 5–10% as at “high risk”, and ≥10% as at “very high risk” [18, 19]. When converted to RRs, these correspond to RR categories of <2.0, 2.0–2.9, 2.9–6.0 and ≥6.0, respectively, based on an estimated population EOC risk between ages 20 to 80 of 1.75%. These RRs are equivalent to 10-year EOC absolute risk categories of <1%, 1–1.4%, 1.4–3% and ≥3% (Supplementary Materials). We also assessed the model sensitivity and specificity using different risk thresholds. All statistical analysis was performed in R (version 4.2.2) [20].

## Results

A total of 199,429 women were included in the analysis. Among these, 733 women developed EOC (0.39%) within the 10-year risk prediction period. Characteristics of eligible participants at baseline are summarised in Table 1. The EOC incidence rates estimated in the UK Biobank for each age group were in line with the UK population incidences [21] (Supplementary Fig. 2).

**Table 1:** Summary of characteristics of 199,429 eligible participants at baseline.

Probands		Unaffected women	Incident EOC patients
199,429		198,656	773
**Variables**	**Category**	***N*** **(%)**	***N*** **(%)**
PVs in six major genes	*BRCA1*	83 (0.04)	7 (0.91)
	*BRCA2*	405 (0.20)	24 (3.10)
	*PALB2*	277 (0.14)	3 (0.39)
	*RAD51C*	52 (0.03)	3 (0.39)
	*RAD51D*	63 (0.03)	2 (0.26)
	*BRIP1*	309 (0.16)	5 (0.65)
	Missing	13,515 (6.80)	50 (6.47)
Parity	Nulliparous	37,498 (18.88)	176 (22.77)
	1 birth	26,373 (13.28)	101 (13.07)
	2 births	88,274 (44.44)	332 (42.95)
	>2 births	46,386 (23.35)	164 (21.22)
	Missing	125 (0.06)	0 (0)
Menopausal hormone therapy use	Current C-type	32,588 (16.40)	146 (18.89)
	Current E-type	117 (0.06)	4 (0.52)
	Former	36,270 (18.26)	181 (23.42)
	Never	129,163 (65.02)	440 (56.92)
	Missing	518 (0.26)	2 (0.26)
Menopause	Yes	123,516 (62.18)	559 (72.32)
	Missing	1 (0)	0 (0)
Age at start of risk prediction	(40, 45)	16,015 (8.07)	25 (3.23)
	(45, 50)	27,097 (13.65)	73 (9.44)
	(50, 55)	31,643 (15.94)	99 (12.81)
	(55, 60)	35,807 (18.03)	126 (16.30)
	(60, 65)	46,992 (23.67)	207 (26.78)
	(65, 70)	35,644 (17.95)	216 (27.94)
	≥70	5458 (2.75)	27 (3.49)
	Missing	0 (0)	0 (0)
	Mean	57	59
	SD	7.97	7.37
Follow-up (years)	Mean	10	6
	SD	2	3
Standardised PRS	Mean	0.84	1.13
	SD	1.02	1.04
	Missing	0 (0)	0 (0)
Duration of oral contraceptive use	Never or <1	6528 (3.29)	28 (3.62)
	1–4	30,215 (15.22)	125 (16.17)
	5–9	36,359 (18.31)	130 (16.82)
	10-14	31,659 (15.94)	100 (12.94)
	≥15	41,929 (21.12)	106 (13.71)
	Missing	51,966 (26.16)	265 (34.28)
	Mean	10.87	9.34
	SD	8.04	7.59
Tubal ligation	Yes	1033 (0.52)	4 (0.52)
	Missing	0 (0)	0 (0)
Endometriosis	Yes	2007 (1.01)	9 (1.16)
	Missing	0 (0)	0 (0)
Height (cm)	Mean	162.7	162.2
	SD	6.3	6.3
	Missing	391 (0.19)	1 (0.13)
BMI	<18.5	1512 (0.76)	3 (0.48)
	(18.5, 25)	80,143 (40.34)	289 (37.25)
	(25, 30)	72,107 (36.30)	288 (38.81)
	≥30	44,311 (22.31)	190 (24.58)
	Missing	583 (0.29)	3 (0.48)

*MHT* menopause hormone therapy, *PRS* polygenic risk score, *BMI* body mass index, *S*D standard deviation.

### Model discrimination and calibration

**Fig. 1:**
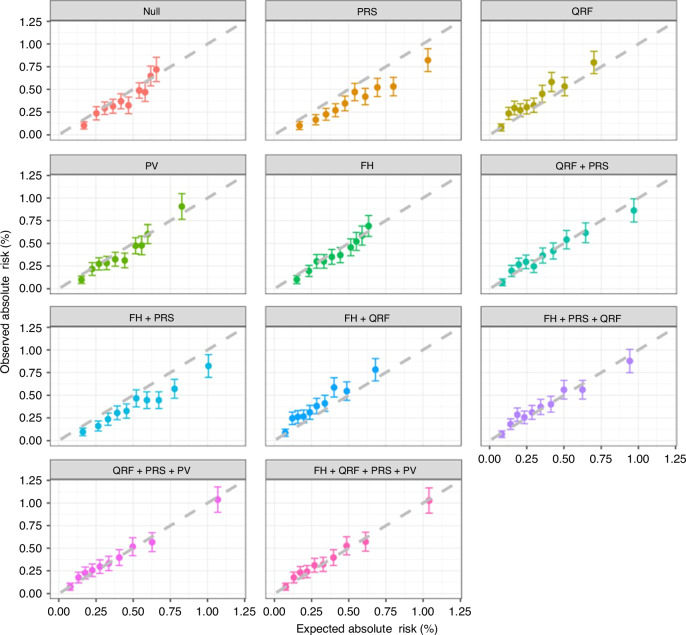
Observed and predicted 10-year EOC risks under individual or different combinations of risk factors*. *Women were grouped into deciles based on their predicted risks. Each dot represents the mean observed and predicted risk in the decile and the error bar represents the 95% confidence intervals. The dashed line is the reference line with slope equals to 1, on which the observed risk equals to the predicted risk. When the confidence interval intersects with the reference line, the predicted risk in that decile is not significantly different from the observed risk. If the confidence interval of a decile deviates from the reference line, there is a suggestion of either overprediction (below the line) or underprediction (above the line) of EOC risks by the BOADICEA model. Null age-only model, PRS polygenic risk scores, PV pathogenic variants, QRF questionnaire-based risk factors, FH family history.

When the PRS, QRFs and PVs were each considered individually in the model in addition to age, the discriminative ability was similar (AUCs = 0.64–0.65 vs 0.61 for age alone; Table 2). There was some evidence of systematic overprediction of risks when considering PRS alone across the deciles of predicted risks (E/O = 1.38, 95% CI: 1.29–1.49; calibration slope = 1.06, 95% CI: 1.05–1.08). The model considering QRF alone underestimated the EOC risk overall (E/O = 0.80, 95% CI: 0.75–0.86), but this was driven by some deciles. The calibration slope was only slightly lower than 1 (calibration slope = 0.96, 95% CI: 0.94–0.97) (Table 2 and Fig. 1). The calibration slope when considering PVs alone was 1.02 (95% CI: 1.00–1.03) (Table 2 and Fig. 1). As expected, when QRF, PRS and PV were jointly considered in the model, the discriminative ability of the model was greater than any of the individual factors (AUC = 0.68, 95% CI: 0.66–0.70). This model was well calibrated (E/O = 0.99, 95% CI: 0.92–1.06; calibration slope = 1.00, 95% CI: 0.98–1.01, Table 2 and Fig. 1). The addition of FH of breast and prostate cancer to age also improved the discrimination (AUC = 0.63), but addition of FH to the model including QRF + PRS + PV model did not further improve the discriminative ability (Table 2 and Fig. 1). However, for completeness and since FH is included in practice, we considered the full model, including FH in all subsequent analyses. The patterns were similar when using Harrell’s C-index as the measure of discrimination.

**Table 2:** Calibration and discrimination of 10-year risk prediction of EOC by the BOADICEA model using individual or different risk factor combinations in the entire cohort, by age group and in pathogenic variant (PV) carriers.

Model	AUC (95% CI)	Harrell’s C-index (95% CI)	E/O (95% CI)	Calibration slope (95% CI)
Entire cohort (*N* = 199,429)				
Null (age-only)	0.61 (0.59, 0.63)	0.63 (0.61, 0.65)	1.11 (1.03, 1.19)	1.02 (1.00, 1.04)
PRS	0.65 (0.63, 0.67)	0.65 (0.63, 0.67)	1.38 (1.29, 1.49)	1.06 (1.05, 1.08)
QRF	0.64 (0.62, 0.66)	0.63 (0.62, 0.66)	0.80 (0.75, 0.86)	0.96 (0.94, 0.97)
PV	0.64 (0.62, 0.66)	0.65 (0.63, 0.67)	1.08 (1.01, 1.16)	1.02 (1.00, 1.03)
FH	0.63 (0.61, 0.65)	0.63 (0.60, 0.64)	1.07 (0.99, 1.14)	1.01 (1.00, 1.03)
FH + QRF	0.64 (0.62, 0.66)	0.63 (0.61, 0.66)	0.77 (0.72, 0.83)	0.95 (0.94, 0.97)
FH + PRS	0.65 (0.63, 0.67)	0.65 (0.62, 0.66)	1.34 (1.25, 1.43)	1.06 (1.04, 1.07)
QRF + PRS	0.66 (0.64, 0.68)	0.65 (0.63, 0.67)	1.00 (0.94, 1.08)	1.00 (0.98, 1.01)
FH + PRS + QRF	0.66 (0.64, 0.68)	0.65 (0.63, 0.67)	0.97 (0.90, 1.04)	0.99 (0.98, 1.01)
QRF + PRS + PV	0.68 (0.66, 0.70)	0.68 (0.66, 0.70)	0.99 (0.92, 1.06)	1.00 (0.98, 1.01)
FH + QRF + PRS + PV	0.68 (0.66, 0.70)	0.68 (0.66, 0.69)	0.96 (0.90, 1.03)	0.99 (0.98, 1.01)
Women < 60 years old (*N* = 110,885)				
FH + QRF + PRS + PV	0.66 (0.63, 0.69)	0.66 (0.62, 0.69)	0.87 (0.78, 0.97)	0.98 (0.95, 1.00)
Women ≥ 60 years (*N* = 88,544)				
FH + QRF + PRS + PV	0.65 (0.63, 0.68)	0.64 (0.60, 0.66)	1.03 (0.94, 1.13)	1.01 (0.98, 1.03)
PV carriers (*N* = 1231)				
PV	0.73 (0.67, 0.80)	0.71 (0.64, 0.79)	0.90 (0.67, 1.21)	0.96 (0.85, 1.07)
PV + QRF + PRS	0.76 (0.69, 0.82)	0.73 (0.66, 0.80)	0.77 (0.57, 1.04)	0.91 (0.80, 1.02)
PV + QRF + PRS + FH	0.76 (0.69, 0.82)	0.73 (0.64, 0.80)	0.73 (0.54, 0.98)	0.90 (0.79, 1.00)

*PRS* polygenic risk scores, *QRF* questionnaire-based risk factors, *PV* pathogenic variants, *FH* family history, *E* expected number of EOCs in the 10-year risk prediction period, *O* observed number of EOCs in the 10-year risk prediction period.

### Model performance by age group

**Fig. 2:**
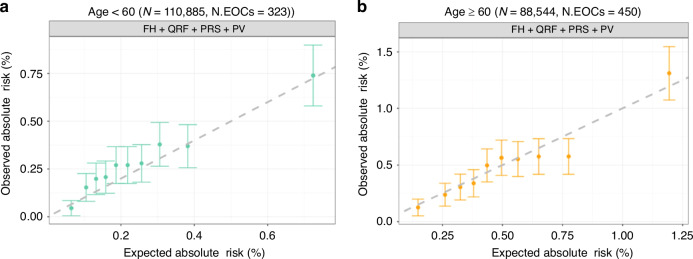
Observed and predicted 10-year EOC risks by age under the full model. **a** Age <60 (*N* = 110,885); **b** age ≥60 (*N* = 88,544). Women were grouped into deciles based on their predicted risks. Each dot represents the mean observed and predicted risk in the decile and error bar represents the 95% confidence intervals.

There were 110,885 women younger than age 60 years (323 incident EOC patients) and 88,544 women aged 60 years or older (450 incident EOC patients). When considering the full model (QRF, PRS, PV and FH of breast/prostate cancer), there was some underprediction of the overall risk (E/O = 0.87, 95% CI: 0.78–0.97) in the <60 years age group. However, the observed risks were consistent with those predicted in all deciles and the calibration slope was consistent with 1 (slope = 0.98, 95% CI: 0.95–1.00). The model was well calibrated in the ≥60 years age group (E/O = 1.03, 95% CI: 0.94–1.13; calibration slope = 1.01, 95% CI: 0.98–1.03; Fig. 2). The discriminative ability was similar between the two age groups with AUCs of 0.65–0.66 (Table 2).

### Model performance in PV carriers

**Fig. 3:**
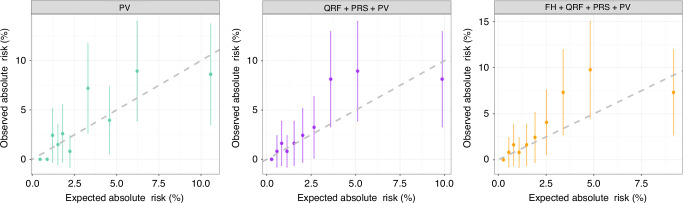
Observed and predicted 10-year EOC risks in pathogenic variant carriers (*N* = 1231) under the model considering PV only, PV + PRS + QRF and PV + PRS + QRF + FH. Women were grouped into deciles based on their predicted risks. Each dot represents the mean observed and predicted risk in the decile, and the error bar represents the 95% CIs.

A total of 1231 women carried PVs in one of the six EOC susceptibility genes. Among those, 44 developed EOC during the 10-year risk prediction period. Compared to the model considering PV alone, the addition of QRF and PRS provided a wider range of risks. The later model predicted risks that were in line with the observed risks in deciles of predicted risk, but the observed risks in each decile were associated with wide confidence intervals due to the small number of incident cancers. For this model, the overall E/O was 0.77 (95% CI: 0.57–1.04) and the calibration slope was 0.91 (95% CI: 0.80–1.02) with some evidence of underestimation in the 8th and 9th deciles (Fig. 3). The full model, that also included FH of breast and prostate cancer (but not EOC) showed similar patterns (Fig. 3). Compared with the model considering PV only, the full model improved the AUC from 0.73 (95% CI: 0.67–0.80) to 0.76 (95% CI: 0.69–0.82; Table 2).

### Risk classification

The 10-year risk thresholds for defining the four risk groups, using the age-dependent and the age-independent approach, are shown in Supplementary Fig. 3. The model considering only FH of breast and prostate cancer or FH and QRFs identified 0% of women with RR of 2.9 or greater or 10-year risk of 1.4% or greater. The full model considering FH, QRF, PRS and PV classified 97.7%, 1.7%, 0.4% and 0.2% women in the RR < 2.0, 2.0–2.9, 2.9–6.0 and ≥6.0 categories and 96.6%, 2.5%, 0.7% and 0.2% women in 10-year risk <1%, 1–1.4%, 1.4–3%, and ≥3% respectively (Supplementary Table 1). When defining individuals in the top 10%, 30% and 50% of the predicted risk distribution as at risk, it identified 19.8%, 41.8%, and 64.7% of incident EOC patients (Supplementary Table 2).

With age-dependent thresholds, the full model classified 1.1%, 0.2% and 0.3% of women younger than age 60 in the 2.0 ≤ RR < 2.9, 2.9 ≤ RR < 6.0 and RR ≥ 6.0 categories, identifying 2.8%, 0.9% and 7.1% of incident EOC patients occurring in the 10-year period, respectively. Whereas among women aged 60 years or older, 2.5%, 0.5% and 0.1% were in the 2.0 ≤ RR < 2.9, 2.9 ≤ RR < 6.0 and RR ≥ 6.0 categories, accounting for 4.5%, 2% and 1.3% of incident EOC patients respectively (Supplementary Table 3). Using the age-independent 10-year risk thresholds, the model classified a larger proportion of higher-risk women in the age ≥60 than age<60 group, with 5.1%, 1.4% and 0.2% of women aged 60 years or older in 1%≤risk<1.4%, 1.4% ≤ risk <3% and risk ≥3% categories, identifying 11.5%, 2.7% and 2.0% incident EOC patients respectively (Supplementary Table 3).

In the PV carriers, using RR thresholds, the model considering PV only classified 13.6%, 42.1% and 44.4% carriers in the 2.0 ≤ RR < 2.9, 2.9 ≤ RR < 6.0 and RR ≥ 6.0 categories, identifying 2.3%, 31.8% and 65.9% of incident EOC occurred respectively. In contrast, the full model considering all factors classified 17.4%, 29.4% and 36.4% women in these categories, identifying 4.6%, 22.7% and 65.9% of incident EOC patients. Similar outcomes were observed using age-independent 10-year risk thresholds (Supplementary Tables 4 and  5).

## Discussion

This is the first study to validate the 10-year EOC risk predictions provided by the BOADICEA model [11], using data from a large independent prospective cohort and including FH, PRS, QRF and PV information. The results show that the full model is well calibrated both overall and in deciles of predicted risk. We also show that the full model has better discriminative ability than models based on any of the individual component factors. The analysis uses the latest version of BOADICEA that is implemented in the CanRisk tool (www.canrisk.org) and is widely used in clinical practice.

One previous study validated an earlier version of BOADICEA [13] in predicting 5-year EOC risk in a small cohort of 1,961 women from the UKCTOCS study. This analysis incorporated FH, a 15-SNP PRS and a subset of QRFs [13]. It reported that BOADICEA was well calibrated and estimated an AUC of 0.61 (95% CI: 0.58–0.64). Due to small differences in model versions, variations in prediction time horizons, the use of different populations, and differences in risk factor availability, direct comparisons of validation results across these two studies are not possible. This is the first validation study to assess the inclusion of the 36-SNP PRS and PV status in the six EOC susceptibility genes. The inclusion of each of these led to improvement in model discriminative ability. Specifically, the addition of PV information to the other factors further improved the model AUC from 0.66 (95% CI: 0.64–0.68) to 0.68 (95% CI: 0.66–0.70), identified additional 0.4% of women with RR of 2.9 or greater (Supplementary Table 2).

When assessed by age group, the discriminatory ability was similar in women younger or older than age 60 years. The model appeared well calibrated in both age groups by most measurements, with the exception of some underprediction of the overall risk in women younger than 60 years.

The frequency of PV carriers in the study sample (Table 1) is lower than reported in previous case-control studies, particularly for *BRCA1* [22]. However, this is most likely due to the fact that the study participants were 40 years old or older at recruitment and that our sample included women without a previous cancer diagnosis at recruitment. Given the known high cancer risks associated with *BRCA1* PVs (including under age 40), this would lead lower number of PV carriers in the sample.

Among PV carriers, the inclusion of QRF and PRS improved the model discriminative ability from AUC = 0.73 (95% CI: 0.67–0.80) to 0.76 (95% CI: 0.69–0.82) and improved the model calibration, especially for low-risk women. As the clinical utility of a model for prevention and early detection depends on its ability in risk stratification, the clinical significance of this improvement is evident. However, there was some underestimation of EOC risks for PV carriers in the 8th and 9th deciles of predicted risks. Although this was not significant, this may potentially be driven by the lack of EOC FH information in the UK Biobank, discussed below. Only 44 PV carriers developed incident EOC in the present study, thus the power to evaluate model calibration was more limited in PV carriers and the power to assess whether the improvement in discriminative ability by including QRF and PRS was significant was limited [23]. Larger studies with longer follow-up will be required to assess the model performance more reliably among PV carriers.

We considered risk stratification based on either absolute or relative risk. The former may be more appropriate when considering decisions on short-term inventions like screening, but results in women at younger ages being classified at low risk who would be high risk when older; thus stratification based on relative risk may be more relevant for interventions such as risk-reducing surgery. As expected, and notwithstanding the modest improvement in the AUC, addition of PVs makes a large difference to the proportion of women classified at ≥2.9-fold risk (0.2–0.6%, proportion of incident cases from 0.4 to 5.3%, Supplementary Table 1), underlining that PV detection is key to detecting women at high long term EOC risk. Among PV carriers, in contrast, the addition of the other factors in the full model markedly increases the proportion of women classified at <2.9-fold risk to more than a third (13.6–34.2%). These results illustrate how incorporating all the risk factors can better define the population most appropriate for interventions, however defining optimal risk thresholds requires careful consideration of the benefits and harms of specific interventions [24]. However, the results indicate that risk stratification for EOC remains challenging at the population level and use of the multifactorial model is likely to be more useful in family clinic or clinical genetics settings in the first instance to facilitate personalised decision-making in clinical EOC risk management.

Strengths of the present study include the very large sample size of the general population and the wide range of risk factor information available. The overall missing risk factor proportion was low in the dataset with <7% missing PV status and <0.3% missing any of the QRF measures, with the exception of duration of oral contraceptive use (3.3%). Previous research has shown that the UK Biobank cohort is subject to “healthy volunteer bias”, in which the participants tend to have healthier lifestyles and had fewer self-reported health conditions compared with the general population [25]. However, the age-specific EOC incidence rates in UK Biobank are very similar to the population incidences (Supplementary Fig. 2), suggesting that healthy volunteer bias is unlikely to be a major factor for EOC.

This study has some limitations. An important limitation is that, while FH of breast and prostate cancer was available, FH of EOC was not collected in UK Biobank. Although BOADICEA incorporates the associations between PVs and breast and prostate cancer, this is of limited value in predicting the risk of EOC once PV status is known. In addition, pedigrees were constructed based on a series of assumptions (Supplementary Materials) including for ages at cancer diagnoses and ages of family members, which were not confirmed in the study. These factors likely explain why the inclusion of FH information did not show a significant improvement in the model performance compared to a previous study [13]. Other cohorts with detailed FH information could provide better validation of the FH component of the model. Second, a total of 18,248 women (9.2%) were censored as unaffected before reaching the end of the 10-year follow-up. As the EOC risks were predicted to the censored age for these women, the estimation of AUC might be upwardly biased. To account for this, we also calculated the Harrell’s C-index which takes account of the time to the event into consideration [16]. The results were very similar to the AUC with no differences in the conclusions. Third, only women of European ancestry were included in this validation study due to the very small number of EOC cases (*N* = 38) in UK Biobank among women of all other ancestries. Future validations should be performed to assess the EOC risk predictions in other populations. Fourth, individual risk predictions may be associated with uncertainty due to missing risk factor information, the precision in the model input parameters and underlying model uncertainty. However, we have not assessed these sources of risk uncertainty in the current study. Finally, BOADICEA does not currently include the effects of rare PV variants in other cancer susceptibility genes, such as mismatch repair genes [22] so we were not able to assess the model performance in this group of PV carriers.

BOADICEA as implemented in the CanRisk tool (www.canrisk.org) has recently been endorsed by the National Institutes of Health and Care Excellence (NICE) guidance on ovarian cancer risk management [26]. The risk-based recommendations include primary prevention with oral contraceptives; discussing and offering risk-reducing salpingo-oophorectomy and informing the timing of the surgery; and increased surveillance with CA-125. The results in this study suggest that BOADICEA provides valid 10-year EOC risks, that can form the basis for shared decision-making in managing ovarian cancer risk.

## Supplementary information

Supplemental Material

## Data Availability

Requests for UK Biobank should be made to the UK Biobank Access Management Team.
