# Supplementary material for: Validation of the BOADICEA model for epithelial tubo-ovarian cancer risk prediction in UK Biobank
Source: Br J Cancer. 2024 Sep 18;131(9):1473–9. doi: 10.1038/s41416-024-02851-z (PMC11519606; doi:10.1038/s41416-024-02851-z)
Supplement: Supplementary file 1 — Supplemental Material [file 41416_2024_2851_MOESM1_ESM.docx]

Supplementary Materials

# Pedigree construction

The pedigrees were constructed and taken into account for all women except for those reported to be adopted. For each participant, the UK Biobank collected data on the ages of parents, the number of biological brothers, the number of biological sisters and self-reported breast and prostate cancer status of the mother, father, and siblings (any) separately. If the proband has many siblings, it is unknown who developed cancer.

Based on this summarized family history information, we constructed family pedigrees for each individual under the following assumptions:

(a)  The age of cancer diagnosis for family members was assumed to be (1) the age of death if the affected family member was deceased or (2) the last follow-up age.

(b)  The last follow-up age of siblings was assumed to be the same as the age of the proband. The last follow-up age of parents is the age of parents collected at baseline.

(d)  Since the number of affected siblings was not available in the UK Biobank, we assumed that only one sibling was affected if there were any affected siblings.

(e)  If sibling was diagnosed with breast cancer, we assumed that the diagnosis occurred is in a sister. Only when the proband did not have any sisters, we assumed that the breast cancer occurred in a brother.

# Genetic data

The UK Biobank samples were genotyped using the Affymetrix UK BiLEVE Axiom array and Affymetrix UK Biobank Axiom array. Imputation was performed using SHAPEIT3 and IMPUTE3.1820 to the combined 1000 Genomes Project v.3 and UK10K reference panels. The genotyping and imputed data were used to derive the 36-SNP polygenic risk score (PRS) and was standardised using a mean of -0.259 and standard deviation of 0.315 (1).

Pathogenic variant carriers in *BRCA1*, *BRCA2*, *RAD51C*, *RAD51D*, *BRIP1* and *PALB2* were identified using UK Biobank whole exome sequencing data. QC metrics were applied to Variant Call Format files as described by Gardner et al, including genotype level filters for depth and genotype quality (2). Other filters including samples with disagreement between genetically determined and self-reported sex, excess relatives etc were applied as described elsewhere (3). The Ensembl Variant Effect Predictor (VEP) was used to annotate variants, and protein truncating variants were considered here. Protein truncating variants in the last exon of each gene and the last 50 bp of the penultimate exon were excluded as these are generally predicted to escape Nonsense-Mediated mRNA Decay (NMD). For each gene, a burden variable was then created where genotypes were collapsed to a 0/1 variable based on whether samples carried a pathogenic variant.

# Risk thresholds

Manchanda et al’s studies suggested categorising women with a *lifetime risk* of epithelial ovarian cancer (EOC) from age 20 to 80 as follows: less than 3.5% considered at low risk, 3.5-5% as at average risk, 5-10% as at high risk, and 10% or greater as at very high risk (3, 4). Assuming a population lifetime EOC risk of 1.75% (which was calculated from age 20 to 80 using BOADICEA, for women born in 1950-1959, when the majority of women in the UK Biobank cohort were born), these categories correspond to relative risk (RR) categories of less than 2.0, 2.0-2.9, 2.9-6.0, and 6.0 or greater respectively using the formula in Pashayan *et al* (5):

$${RR}_{threshold}=\frac{log(1.0-lifetime risk threshold)}{log(1.0-population average risk)}$$

The corresponding 10-year absolute EOC risk thresholds for individuals at age i were calculated as:

$${Risk}_{threshold}\left( i \right)=1.0-{(1-{Risk}_{pop}\left( i \right))}^{RR}$$

where Risk_pop_(i) was the population 10-year EOC risk from age i and was calculated using the BOADICEA. RRs were the calculated relative risk thresholds (i.e. 2.0, 2.9 or 6.0). As a result, the corresponding 10-year absolute EOC risk categories, assuming the median age of 58 years old for women in UK Biobank were calculated as: less than 1%, 1-1.4%, 1.4-3%, and 3% or greater.

Supplementary Figure 1 Flow chart of the selection of participants in the UK Biobank


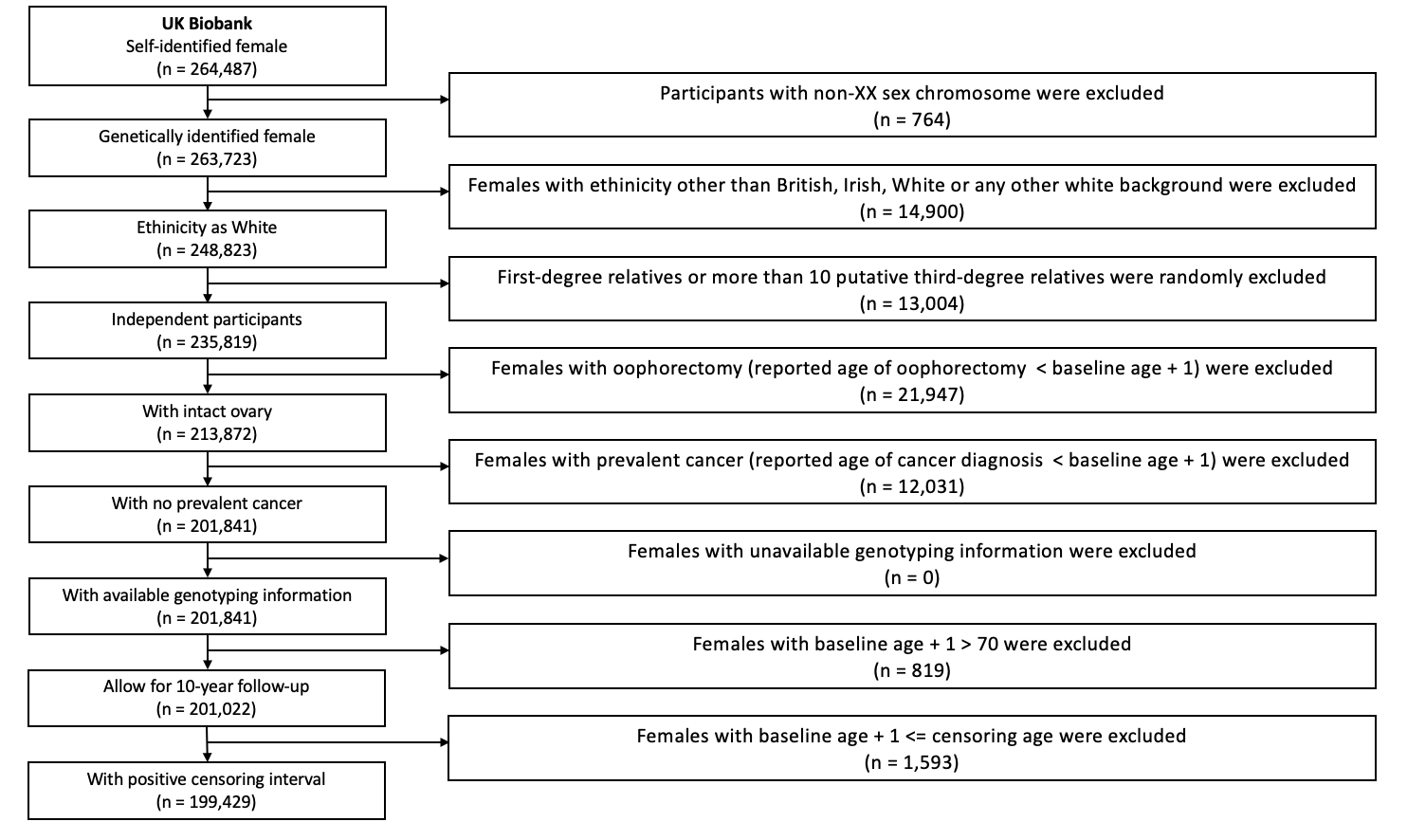


Supplementary Figure 2 Comparison of the age-specific EOC incidence rates per 100,000 women between estimates from UK Biobank (blue line) and Cancer Research UK (CRUK, 2016-2018, red line)***.*** The error bars represent 95% CI estimated for the UK Biobank incidence rates.

**
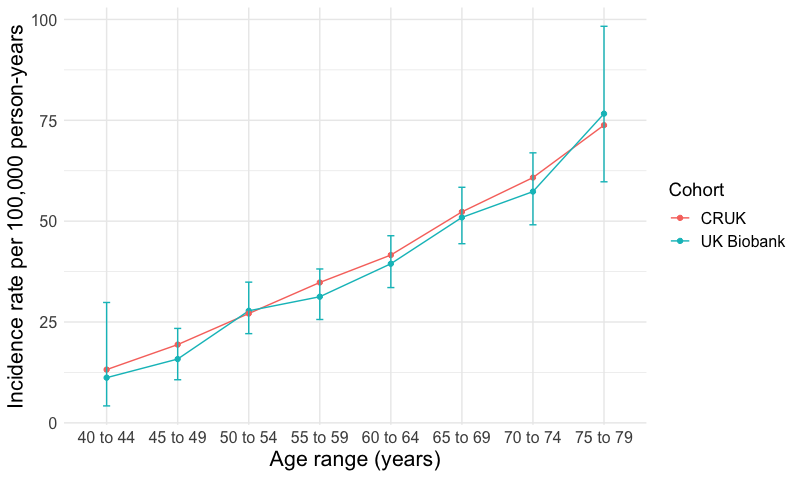
**

Supplementary Figure 3 Age-dependent absolute risk thresholds based on relative risk thresholds (solid line) and age-independent absolute risk thresholds (dashed line) representing 10-year absolute risk of 1%, 1.4% and 3%, which are equivalent to relative risk of 2.0, 2.9 and 6.0 at the median age of 58 years for women In the UK Biobank.


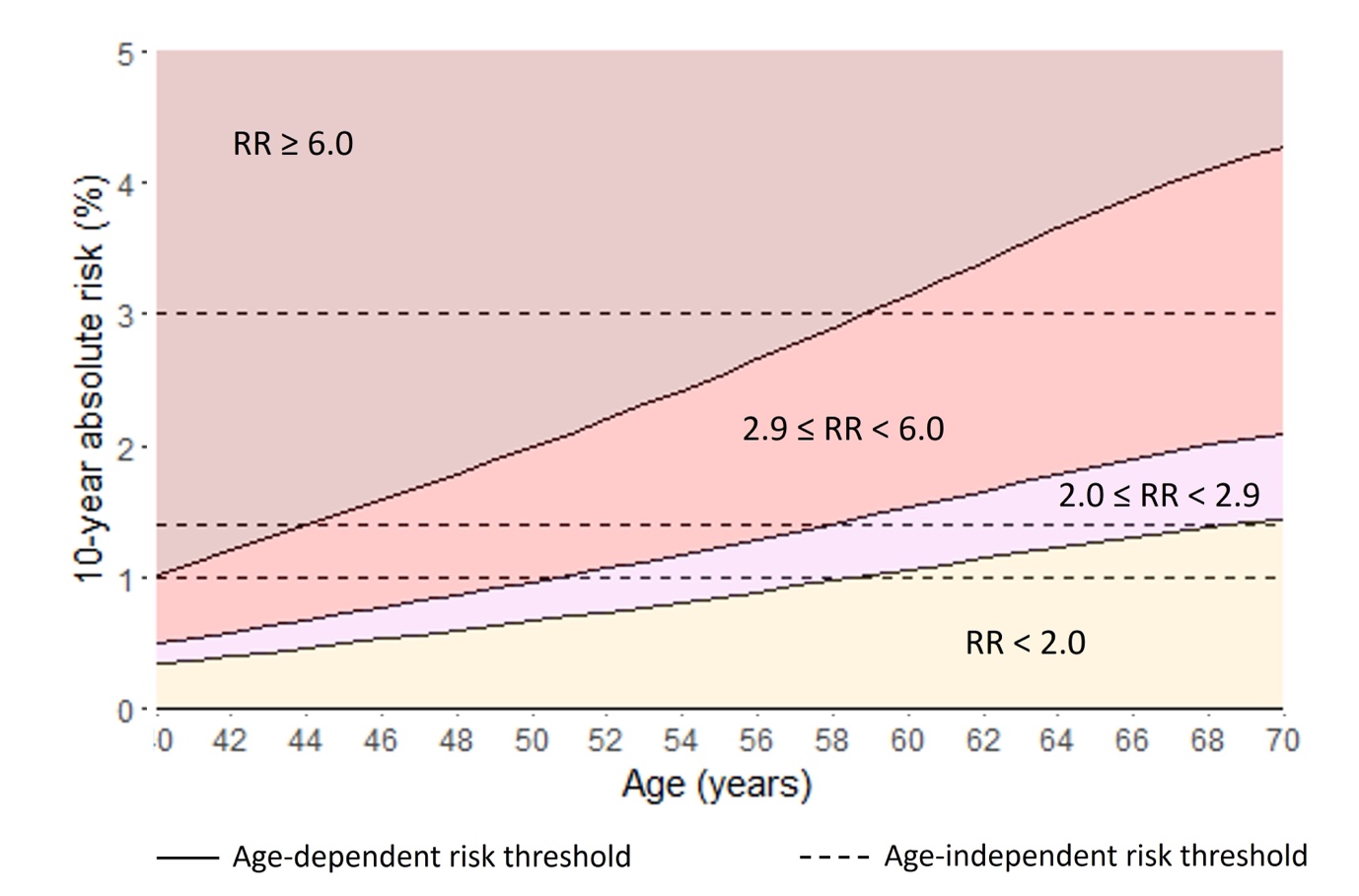


Supplementary Table 1 Summary of risk classification by different combinations of risk factors using the relative risk (RR) thresholds and absolute 10-year EOC risk thresholds in the entire UK Biobank cohort (N=199,429, Number of EOC cases=773).

|  | Population (%) | | | | Incident EOC patients (%) | | | | |
| --- | --- | --- | --- | --- | --- | --- | --- | --- | --- |
|  | **FH** | **FH+QRF** | **FH+PRS+QRF** | **FH+PRS+QRF+PV** | **FH** | **FH+QRF** | **FH+PRS+QRF** | **FH+PRS+QRF+PV** | |
| *RR thresholds* | | | | | | | | | |
| RR<2.0 | 199327  (99.9) | 199240  (99.9) | 194981 (97.8) | 194834  (97.7) | 773  (100) | 771  (99.7) | 740  (95.7) | | 703  (90.9) |
| 2.0 ≤ RR < 2.9 | 80  (0) | 177 (0.1) | 3988  (2) | 3435  (1.7) | 0  (0) | 2  (0.3) | 30  (3.9) | | 29  (3.8) |
| 2.9 ≤ RR < 6.0 | 22  (0) | 11  (0) | 457  (0.2) | 711  (0.4) | 0  (0) | 0  (0) | 3  (0.4) | | 12  (1.5) |
| RR ≥ 6.0 | 0  (0) | 1  (0) | 3  (0) | 449  (0.2) | 0  (0) | 0  (0) | 0  (0) | | 29  (3.8) |
| *Absolute 10-year EOC risk thresholds* | | | | | | | | | |
| risk < 1% | 199410  (100) | 198539  (99.6) | 193030  (96.8) | 192719  (96.6) | 773  (0) | 770  (99.6) | 712  (92.1) | | 673  (87.1) |
| 1% ≤ risk < 1.4% | 14  (0) | 862  (0.4) | 5228  (2.6) | 4898  (2.5) | 0  (0) | 3  (0.4) | 53  (6.9) | | 55  (7.1) |
| 1.4% ≤ risk < 3% | 5  (0) | 28  (0) | 1169  (0.6) | 1391  (0.7) | 0  (0) | 0  (0) | 8  (1.0) | | 15  (1.9) |
| risk ≥ 3% | 0  (0) | 0  (0) | 2  (0) | 421  (0.2) | 0  (0) | 0  (0) | 0  (0) | | 30  (3.9) |

Supplementary Table 2 The proportion of incident EOC patients and the proportion of healthy women identified in the entire UK Biobank cohort using a series of thresholds based on the full model.

| Thresholds | Proportion of incident EOC patients with risks above the threshold (%, 95% CI) | Proportion of healthy women with risks below the threshold (%, 95% CI) |
| --- | --- | --- |
| Top 5% of predicted risk | 8.5 (6.7, 10.7) | 94.3 (94.2, 94.4) |
| Top 10% of predicted risk | 19.8 (17.0, 22.8) | 87.5 (87.3, 87.6) |
| Top 15% of predicted risk | 26.1 (23.1, 29.4) | 83.5 (83.3, 83.7) |
| Top 20% of predicted risk | 31.4 (28.2, 34.8) | 79.3 (79.1, 79.5) |
| Top 25% of predicted risk | 35.7 (32.3, 39.2) | 74.8 (74.6, 75.0) |
| Top 30% of predicted risk | 41.8 (38.3, 45.4) | 69.8 (69.6, 70.0) |
| Top 35% of predicted risk | 48.8 (45.2, 52.4) | 64.6 (64.4, 64.8) |
| Top 40% of predicted risk | 53.7 (50.1, 57.2) | 59.9 (59.7, 60.1) |
| Top 45% of predicted risk | 59.5 (56.0, 63.0) | 54.4 (54.2, 54.6) |
| Top 50% of predicted risk | 64.7 (61.2, 68.1) | 48.1 (47.9, 48.4) |
| RR ≥ 2.0 | 9.1 (7.1, 11.3) | 97.7 (97.7, 97.8) |
| RR ≥ 2.9 | 5.3 (3.8, 7.1) | 99.4 (99.4, 99.5) |
| RR ≥ 6.0 | 3.8 (2.5, 5.3) | 99.8 (99.8, 99.8) |
| 10-year absolute risk ≥ 1% | 12.9 (10.7, 15.5) | 96.7 (96.6, 96.8) |
| 10-year absolute risk ≥ 1.4% | 5.8 (4.3, 7.7) | 99.1 (99.1, 99.2) |
| 10-year absolute risk ≥ 3% | 3.9 (2.6, 5.5) | 99.8 (99.8, 99.8) |

Supplementary Table 3 Summary of risk classification by age group under the full model using RR thresholds and absolute 10-year EOC risk thresholds.

| Thresholds | Age < 60 (N = 110,885; EOC cases N = 323) | | Age ≥ 60 (N = 88,544; EOC cases N = 450) | |
| --- | --- | --- | --- | --- |
|  | Population (%) | Incident EOC patients (%) | Population (%) | Incident EOC patients (%) |
| *RR thresholds* | | | | |
| RR < 2.0 | 109,057 (98.4) | 288 (89.2) | 85,777 (96.9) | 415 (92.2) |
| 2.0 ≤ RR < 2.9 | 1,221 (1.1) | 9 (2.8) | 2,214 (2.5) | 20 (4.5) |
| 2.9 ≤ RR < 6.0 | 263 (0.2) | 3 (0.9) | 448 (0.5) | 9 (2.0) |
| RR ≥ 6.0 | 344 (0.3) | 23 (7.1) | 105 (0.1) | 6 (1.3) |
| *Absolute 10-year EOC risk thresholds* | | | | |
| risk < 1% | 110,106(99.3) | 296 (91.7) | 82,613 (93.3) | 377 (83.8) |
| 1% ≤ risk < 1.4% | 348 (0.3) | 3 (0.9) | 4550 (5.1) | 52 (11.5) |
| 1.4% ≤ risk < 3% | 160 (0.2) | 3 (0.9) | 1231 (1.4) | 12 (2.7) |
| risk ≥ 3% | 271 (0.2) | 21 (6.5) | 150 (0.2) | 9 (2.0) |

Supplementary Table 4 Summary of risk classification in 1,231 pathogenic variant (PV) carriers (44 incident EOC cases) under the model considering PV only and the full model considering PV, questionnaire risk factors, polygenic risk score and cancer family history.

|  | Population (%) | | Incident EOC patients (%) | |
| --- | --- | --- | --- | --- |
| Model | **PV** | **Full** | **PV** | **Full** |
| *RR thresholds* | | | | |
| RR < 2.0 | 0 (0) | 207 (16.8) | 0 (0) | 3 (6.8) |
| 2.0 ≤ RR < 2.9 | 167 (13.6) | 214 (17.4) | 1 (2.3) | 2 (4.6)) |
| 2.9 ≤ RR < 6.0 | 518 (42.1) | 362 (29.4) | 14 (31.8) | 10 (22.7) |
| RR ≥ 6.0 | 546 (44.4) | 448 (36.4) | 29 (65.9) | 29 (65.9) |
| *Absolute 10-year EOC risk thresholds* | | | | |
| risk < 1% | 155 (12.6) | 325 (26.4) | 0 (0) | 3 (6.8) |
| 1% ≤ risk < 1.4% | 164 (13.3) | 128 (10.4) | 3 (6.8) | 2 (4.5) |
| 1.4% ≤ risk < 3% | 388 (31.5) | 359 (29.2) | 10 (22.7) | 9 (20.5) |
| risk ≥ 3% | 524 (42.6) | 419 (34.0) | 31 (70.5) | 30 (68.2) |

Supplementary Table 5 Number of pathogenic variant (PV) carriers in each ovarian cancer susceptibility gene by risk category: a) using the model considering PV only;  and b) using the full model considering PV, questionnaire risk factors, polygenic risk score and cancer family history.

| Risk category | Gene | PV only model,  N of PV carriers | Full model,  N of PV carriers |
| --- | --- | --- | --- |
| *RR thresholds* | | | |
| RR < 2.0 | *BRCA1* | 0 | 0 |
|  | *BRCA2* | 0 | 3 |
|  | *PALB2* | 0 | 106 |
|  | *RAD51C* | 0 | 0 |
|  | *RAD51D* | 0 | 0 |
|  | *BRIP1* | 0 | 98 |
| 2.0 ≤ RR < 2.9 | *BRCA1* | 0 | 0 |
|  | *BRCA2* | 0 | 21 |
|  | *PALB2* | 167 | 98 |
|  | *RAD51C* | 0 | 4 |
|  | *RAD51D* | 0 | 2 |
|  | *BRIP1* | 0 | 89 |
| 2.9 ≤ RR < 6.0 | *BRCA1* | 0 | 0 |
|  | *BRCA2* | 77 | 128 |
|  | *PALB2* | 112 | 69 |
|  | *RAD51C* | 9 | 25 |
|  | *RAD51D* | 7 | 26 |
|  | *BRIP1* | 313 | 115 |
| RR ≥ 6.0 | *BRCA1* | 90 | 90 |
|  | *BRCA2* | 352 | 277 |
|  | *PALB2* | 1^a^ | 7 |
|  | *RAD51C* | 46 | 26 |
|  | *RAD51D* | 58 | 37 |
|  | *BRIP1* | 1^b^ | 12 |
| *Absolute 10-year EOC risk thresholds* | | | |
| risk < 1% | *BRCA1* | 0 | 0 |
|  | *BRCA2* | 6 | 23 |
|  | *PALB2* | 91 | 147 |
|  | *RAD51C* | 0 | 4 |
|  | *RAD51D* | 0 | 5 |
|  | *BRIP1* | 58 | 146 |
| 1% ≤ risk < 1.4% | *BRCA1* | 0 | 0 |
|  | *BRCA2* | 13 | 21 |
|  | *PALB2* | 58 | 49 |
|  | *RAD51C* | 6 | 8 |
|  | *RAD51D* | 3 | 4 |
|  | *BRIP1* | 84 | 46 |
| 1.4% ≤ risk < 3% | *BRCA1* | 0 | 0 |
|  | *BRCA2* | 59 | 131 |
|  | *PALB2* | 130 | 79 |
|  | *RAD51C* | 18 | 20 |
|  | *RAD51D* | 10 | 19 |
|  | *BRIP1* | 171 | 110 |
| risk ≥ 3% | *BRCA1* | 90 | 90 |
|  | *BRCA2* | 351 | 254 |
|  | *PALB2* | 1^a^ | 5 |
|  | *RAD51C* | 31 | 23 |
|  | *RAD51D* | 52 | 37 |
|  | *BRIP1* | 1^b^ | 12 |

^a^ Joint *PALB2* and *BRCA2* PV carrier

^b^ Joint *BRIP1* and *RAD51D* PV carrier

# References:

1. Dareng EO, Tyrer JP, Barnes DR, Jones MR, Yang X, Aben KKH, et al. Polygenic risk modeling for prediction of epithelial ovarian cancer risk. Eur J Hum Genet. 2022;30(3):349-62.

2. Gardner EJ, Kentistou KA, Stankovic S, Lockhart S, Wheeler E, Day FR, et al. Damaging missense variants in IGF1R implicate a role for IGF-1 resistance in the aetiology of type 2 diabetes. MedRxiv. 2022.

3. Wilcox N, Dumont M, Gonzalez-Neira A, Carvalho S, Joly Beauparlant C, Crotti M, et al. Exome sequencing identifies breast cancer susceptibility genes and defines the contribution of coding variants to breast cancer risk. Nat Genet. 2023;55(9):1435-9.
